# Inhibition of LncRNAH19 has the effect of anti‐tumour and enhancing sensitivity to Gefitinib and Chemotherapy in Non‐small‐cell lung cancer in vivo

**DOI:** 10.1111/jcmm.15245

**Published:** 2020-04-13

**Authors:** Yaodong Zhou, Yixin Zhang

**Affiliations:** ^1^ Department of Thoracic Surgery Fudan University Shanghai Cancer Center Shanghai China; ^2^ Department of Oncology Shanghai Medical College Fudan University Shanghai China; ^3^ Department of Plastic and Reconstructive Surgery Shanghai Ninth People’s Hospital affiliated to Shanghai Jiaotong University Shanghai China

**Keywords:** Gefitinib, H19, miR‐21, non‐small‐cell lung cancer, PTEN

## Abstract

Lung cancer is one of the most common malignant diseases, which ranked first in both men and women malignancies worldwide. The survival rate of non‐small‐cell lung cancer (NSCLC) has been limited with distant metastasis and shortage of effective chemotherapeutics in recent years. Thus, novel therapeutic strategies for NSCLC are urgently explored. Here, we showed that inhibition of H19 effectively inhibited the progression of NSCLC. Moreover, down‐regulation of H19 treatment significantly enhanced the levels of PTEN and PDCD4, while suppressed the expressions of NFIB in NSCLC. Furthermore, down‐regulation of H19 combined with Gefitinib treatment significantly increased the levels of PTEN and PDCD4, while decreased the expression levels of NFIB. Moreover, the results showed that Gefitinib treatment significantly reduced the shH19‐mediated miR‐21 expression levels. Our results showed that down‐regulation of H19 combined with Gefitinib administration significantly improved the effect of shH19 treatment alone on the progression of NSCLC, which was involved in the activation of PTEN signalling pathway in NSCLC in vivo. Therefore, these findings might indicate a novel molecular mechanism, which could provide a new potential combination of therapeutic method in NSCLC.

## 1 INTRODUCTION

Lung cancer is a common cause of cancer‐related death in worldwide.^1^ In addition, the morbidity of lung cancer has already ranked first in male and female malignancies.^2^ Non‐small‐cell lung cancer (NSCLC) is the most common form, accounting for approximately 80% of lung cancers. Currently, the prognosis of NSCLC has improved owing to advances in surgical resection and early surveillance and diagnosis.^3^, ^4^ However, accompanied with distant metastasis and shortage of effective chemotherapeutics, the relative 5‐year survival ratio of patients with NSCLC was still quite low, which just ranged from 10% to 15%.^5^, ^6^ Therefore, novel therapeutic strategies for NSCLC are urgently needed to improve its survival rate.

LncRNAH19, a paternally imprinted and maternally expressed 2.7 kb gene, locked close to the telomeric region of chromosome 11p15.5.^7^ The regulation of long non‐coding RNA H19 has been studied intensively in various human diseases such as vascular disease, gestational trophoblastic diseases and liver diseases.^8^, ^9^, ^10^ miR‐675, a highly conserved microRNA sequence that situates at exon 1 of the H19 gene, is one of the most foremost transcripts in the H19 locus.^11^ It is well known that H19 is considered as a regulatory RNA, which involves in numerous biological processes of human diseases, ranging from transcriptional and post‐transcriptional regulation to tumour suppression and oncogenesis.^12^, ^13^ Additionally, H19 has been proposed to play vital function in many cancers, including colorectal cancer, gastric cancer, hepatocellular cancer, breast cancer, cervical cancer and lung cancer.^14^, ^15^, ^16^, ^17^, ^18^, ^19^ The previous study has shown that H19 was closely associated with stem cell phenotype in ALDH1‐positive breast cancer and was involved in poor prognosis in triple‐negative breast cancer.^20^ A recent report has found that H19 was up‐regulated in A549 and H1299 lung cancer cells compared with normal lung BEAS‐2B cells and promoted NSCLC progression through STAT3 signalling way.^21^ In addition, another study showed that plasma level of H19 in NSCLC patients was significantly increased, which could be applied as a serological marker for the auxiliary diagnosis of NSCLC.^22^ Although many related miRNAs and signalling pathways are explored in H19‐regulated lung cancer cells, the combination effects between H19 and chemotherapeutic drugs in controlling progression of lung cancer and their tentative mechanisms are urgently needed to solve.^23^, ^24^

To explore the effect and molecular mechanism of H19‐combined chemotherapeutic drugs in NSCLC, we constructed xenograft nude mouse model using A549 NSCLC cells. In this study, we focused on the functional mechanism of shH19‐regulated PTEN signalling pathway of NSCLC in vivo. Here, we showed that shH19 effectively inhibited the tumour growth in NSCLC. Moreover, shH19 combined with Gefitinib administration significantly improved the shH19 treatment alone in controlling NSCLC development, which was involved in the activation of PTEN signalling pathway in NSCLC in vivo. Therefore, our results provided a new potential combination of therapeutic manner in NSCLC.

## 2 MATERIALS AND METHODS

### 2.1 Medicines and regents

shH19 was purchased from Shanghai Integrated Biotech Solutions Co., Ltd. AntagomiR‐21 was acquired from Guangzhou RiboBio Co., Ltd. Lipofectamine 2000 was purchased from Invitrogen. Gefitinib, pemetrexed and cisplatin were purchased from Selleckchem. NFIB antibody was purchased from Abcam. PTEN, PDCD4 and GAPFH antibodies were obtained from Invitrogen. The Haematoxylin‐Eosin (HE) staining kit was purchased from Shanghai Tiangen Technology Co., Ltd. The RT‐PCR kit was purchased from Invitrogen.

### 2.2 Cell culture

A549 cell lines were obtained from Cell Bank of Shanghai JiaoTong University. RPMI‐1640 medium supplemented with penicillin (100 U/mL, Sigma Chemicals) and streptomycin (100 μg/mL, Sigma Chemicals) and 10% foetal bovine serum was used to be in a humidified incubator with 5% CO2 at 37°C.

### 2.3 Animals

Six‐week‐old BALB/c nude mice were provided by the Animal Center of the Shanghai Jiaotong University. All animals were kept in standard laboratory guidelines. The protocols of animal use were complied by Guide for the Care and Use of Laboratory Animals from Shanghai Jiaotong Medical University, and all experimental rules described in this research were approved by the Animal Care Committee of the Shanghai Jiaotong University Affiliated Ninth People's Hospital. Forty‐two nude mice suffering A549 tumour xenografts were divided into seven groups randomly: Control, shH19, antagomiR‐21, shH19 + pemetrexed +cisplatin, shH19 + Gefitinib, pemetrexed + cisplatin and Gefitinib. The volumes of the tumours were measured every week.

### 2.4 RNA extraction and quantitative real‐time polymerase chain reaction analysis

Total RNA was isolated using TRIzol reagent from Tiangen following the manufacturer's instructions. For analysis of miR‐21, H19, PTEN, PDCD4 and NFIB expression, real‐time PCR analyses were performed using TaqMan miR assays (Applied Biosystems) as the manufacturer's instructions. Standard curve was plotted depended on the standard CT values. The ABI StepOne Plus (Applied Biosystems) was used to perform qPCR amplification, and each experiment was performed in triplicate and repeated at least once.

### 2.5 Western blot

Protein was isolated from sodium dodecyl (10%) sulphate‐polyacrylamide gel (SDS‐PAGE) and then transferred to a nitrocellulose membrane (Tiangen). The primary antibodies and secondary antibodies conjugated with horseradish peroxidase (Tiangen) were used to detect the bands. The ultimate results were visualized and recorded by the blotting analysis system (Thermo Scientific).

### 2.6 Haematoxylin‐Eosin staining

The animal tissues were paraffin fixed and embedded blocks of the formalin‐fixed spinal cord specimens. Then, they were subjected to routine HE staining and observed through an optical microscope. All the images were obtained at 400× magnification.

### 2.7 Statistical analysis

The results of the study were expressed as the mean ± SD and repeated at least three times. Group differences were analysed by Student's *t* test, and *P* < .05 was considered statistically significant. All statistical analyses were performed using SPSS 20.0 software (IBM).

## 3 RESULTS

### 3.1 shH19‐inhibited tumour growth of lung cancer in vivo

**FIGURE 1:**
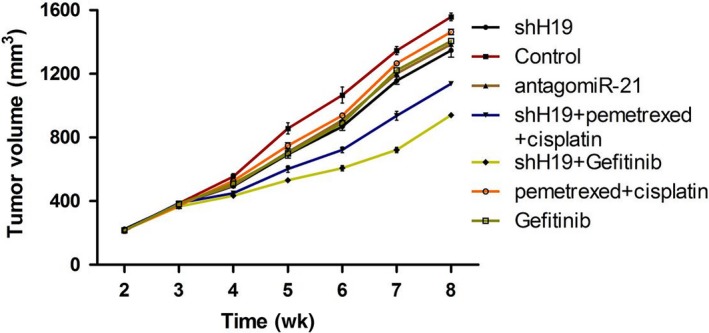
Inhibition of H19‐suppressed tumour growth of lung cancer in vivo. The volumes of the tumours were measured every week

**FIGURE 2:**
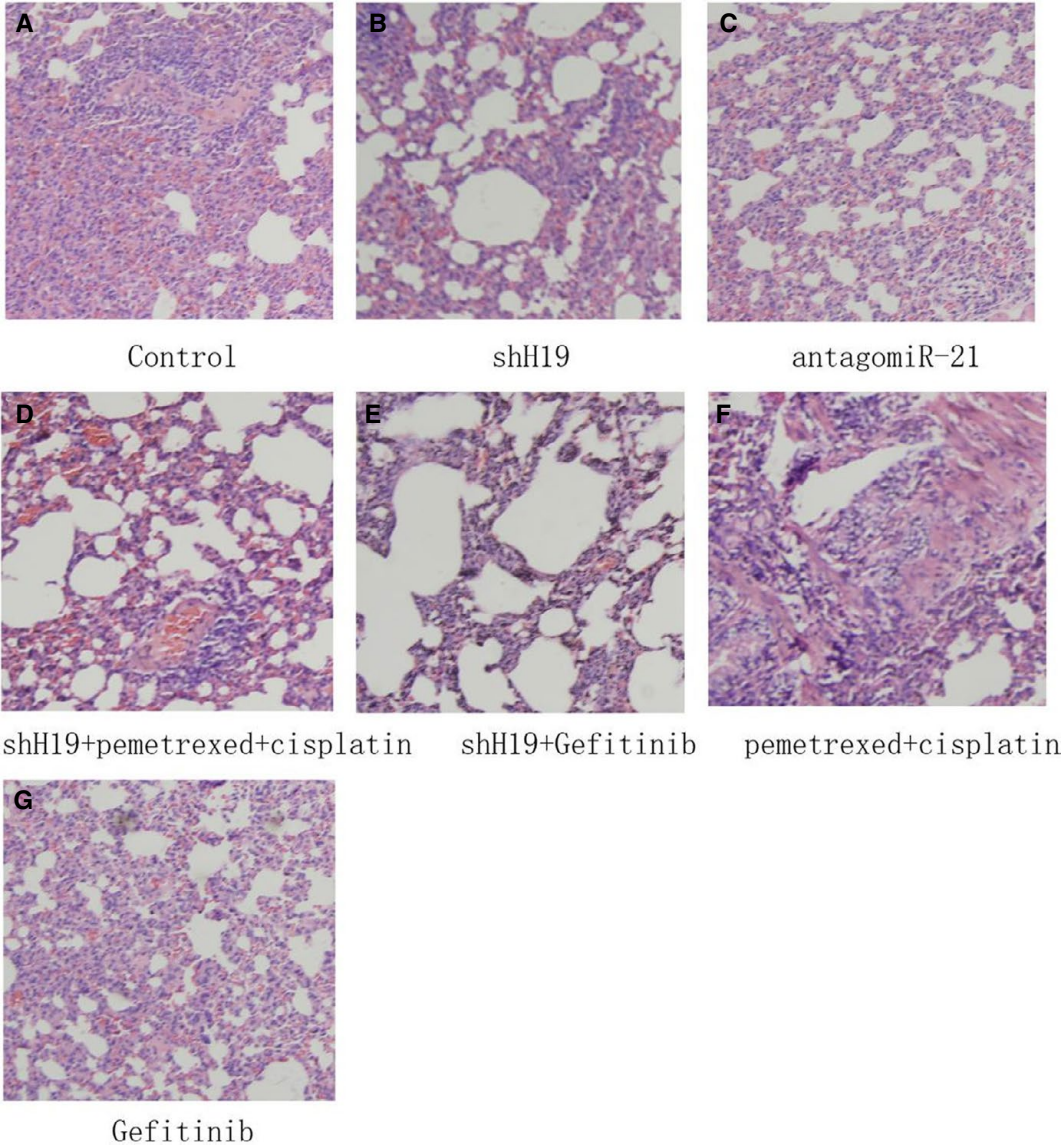
Effects of shH19 combined with Gefitinib or pemetrexed and cisplatin on tumour tissues in lung cancers in vivo. A‐G, HE staining results of tumour tissues in each group

To evaluate the tumour suppressor activation of shH19, we observed whether shH19 could inhibit the progression of tumour growth in a tumour xenograft model. Forty‐two nude mice bearing A549 tumour xenografts were generally divided into seven groups randomly: Control, shH19, antagomiR‐21, shH19 + pemetrexed +cisplatin, shH19 + Gefitinib, pemetrexed + cisplatin and Gefitinib. The volumes of the tumours were measured once a week. As shown in Figure 1, tumour growth was obviously inhibited by the treatment of shH19. In addition, this inhibition effect was most significant by combined treatment with shH19 plus Gefitinib in vivo in comparison with the control group. Moreover, the inhibition effects of Gefitinib or pemetrexed and cisplatin were enhanced by combined treatment with shH19 in lung cancer in vivo. Furthermore, the result showed that antagomiR‐21 administration also effectively suppressed tumour growth of lung cancer in vivo. In order to understand whether shH19 could have an inhibitory effect in tumour tissues of A549 xenografts, HE staining was performed after the mice killed. The results were in accordance with the outcome of tumour volume, and the tumour tissue of xenograft was dramatically restored from mice that received combined treatment with the shH19 and Gefitinib in vivo compared with the control or Gefitinib alone treatment group. In addition, the effects of Gefitinib or pemetrexed plus cisplatin on tumour tissue were also enhanced by combined treatment with shH19 in lung cancer in vivo (Figure 2A‐G).

### 3.2 shH19‐activated PTEN signalling pathway in vivo

**FIGURE 3:**
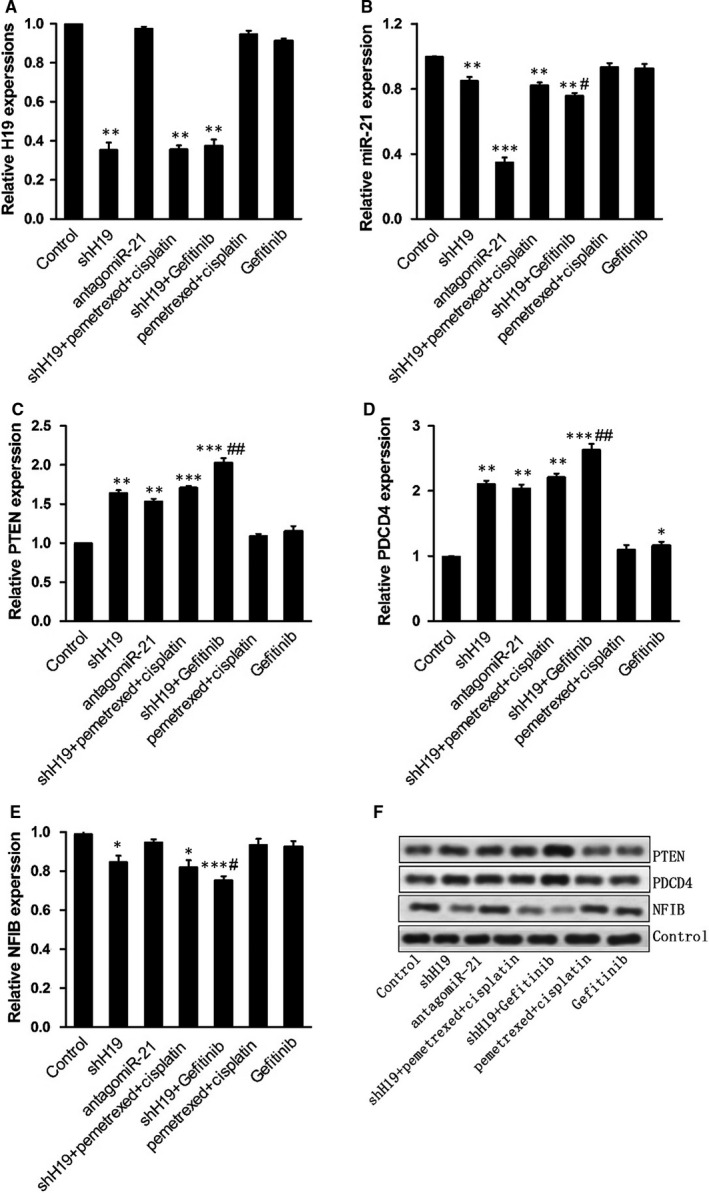
Inhibition of H19‐activated PTEN signalling pathway in vivo*.* A‐E, qPCR detection of miR‐21 and H19 levels and expression of PTEN, PDCD4 and NFIB in tumour tissues. F, Western blot detection of the expression of PTEN, PDCD4 and NFIB in tumour tissues

To further explore the tumour suppressor role of shH19 in downstream signal pathway, PTEN signalling pathway–related gene expression was determined. First, we investigated the expressions of H19 and miR‐21 following different treatments in lung cancer. As expected, shH19 administration significantly inhibited H19 expression levels compared with the control in vivo*.* Moreover, antagomiR‐21 treatment remarkably suppressed miR‐21 expression levels compared with the control in vivo*.* Interestingly, the expression of miR‐21 was obviously inhibited in the treatment of shH19. The results also showed that shH19 combined with Gefitinib treatment significantly reduced the miR‐21 expression levels when compared with shH19 alone treatment, which suggested that Gefitinib might play an important role in suppressing miR‐21 expression in lung cancer in vivo. Furthermore, the results demonstrated that shH19 administration significantly enhanced the expression levels of PTEN and PDCD4, while decreased the expression levels of NFIB in lung cancer. On the other hand, shH19 combined with Gefitinib treatment significantly increased the expression levels of PTEN and PDCD4, while decreased the expression levels of NFIB when compared with shH19 alone treatment, which suggested that Gefitinib partially inhibited PTEN signalling pathway in lung cancer in vivo. In addition, we also found antagomiR‐21 administration obviously enhanced the expression levels of PTEN and PDCD4 in lung cancer. To detect the effect of shH19 on PTEN signalling pathway in NSCLC, the PTEN‐related protein expressions were determined following different treatments in vivo*.* The results were basically in accordance with the mRNA expression levels, and shH19 administration obviously enhanced the protein expression levels of PTEN and PDCD4, while decreased the expression levels of NFIB in NSCLC. Moreover, shH19 combined with Gefitinib treatment obviously increased the protein expression levels of PTEN and PDCD4, while decreased the expression levels of NFIB in lung cancer in vivo. These results suggested that shH19 activated PTEN signalling pathway in lung cancer in vivo (Figure 3A‐F)*.*

## 4 DISCUSSION

Long non‐coding RNA (lncRNA) plays an important role in a multi‐step biological process of tumour, such as cell growth, differentiation, progression and apoptosis.^25^, ^26^ Mounts of evidence demonstrates that lncRNAs were associated with the initiation and development of non‐small‐cell lung cancer.^27^, ^28^ Recently, the study demonstrated that H19 was high expression in non‐small‐cell lung cancer patients.^29^ In the present study, we found that down‐regulation of H19 inhibited the progression of tumour growth in the xenograft model compared with control group. A decrescent small tumour was revealed in the combined treatment of shH19 and Gefitinib compared with down‐regulation of H19 or Gefitinib treatment alone. Moreover, the antagomiR‐21 also showed the inhibition effect on the tumour growth compared with control in vivo. The previous study showed that H19 promoted NSCLC development through STAT3 signalling pathway.^19^ Our results revealed that down‐regulation of H19 significantly inhibited tumour growth and enhanced the effect of pemetrexed and cisplatin or Gefitinib in NSCLC in vivo.

To investigate the tumour suppressor role of shH19 in A549 xenografts, HE staining was performed and the results revealed that combined treatment with the shH19 and Gefitinib dramatically restored tissue morphology of A549 xenografts, which were in accordance with the tumour volume outcome. Moreover, the effects of Gefitinib or pemetrexed and cisplatin on tumour tissue were also enhanced by combined treatment with shH19 in NSCLC in vivo. To further investigate the tumour suppressor role of shH19 in downstream signalling pathway, the expression of PTEN signalling pathway–related genes was explored. The results showed that down‐regulation of H19 treatment significantly enhanced the expression levels of PTEN and PDCD4, while decreased the expression levels of NFIB in NSCLC. On the other hand, down‐regulation of H19 combined with Gefitinib treatment significantly increased the levels of PTEN and PDCD4, while decreased the expression of NFIB when compared with down‐regulation of H19 alone treatment, which suggested that Gefitinib might inhibit PTEN signalling pathway in NSCLC in vivo. In addition, we also found antagomiR‐21 administration obviously enhanced the expression levels of PTEN and PDCD4 in lung cancer. A recent study has shown that valproic acid suppressed the expression of PTEN and p21 through down‐regulation of H19 expression in ovarian A2780 cells, which were in accordance with our results.^30^ These results suggested that down‐regulation of H19 level could activate PTEN signalling pathway in NSCLC in vivo. Interestingly, the expression levels of miR‐21 were obviously inhibited following down‐regulation of H19. There was a previous study about miR‐21, which joined in exosome‐mediated Gefitinib resistance in lung cancer HCC827 cells.^31^ In our research, the results showed that Gefitinib treatment significantly reduced the shH19‐mediated miR‐21 expression levels, which suggested that Gefitinib might play an important role in suppressing miR‐21 expression in NSCLC in vivo.

In conclusion, our study demonstrated that down‐regulation of H19 effectively inhibited the progression of NSCLC in vivo. Moreover, down‐regulation of H19 combined with Gefitinib administration significantly improved down‐regulation of H19 treatment alone in controlling NSCLC development, which participated in the activation of PTEN signalling pathway in NSCLC in vivo. These findings might indicate a novel molecular mechanism, which could provide a new potential combined combination of therapeutic method in NSCLC.

## CONFLICT OF INTEREST

The authors declare that they have no conflict of interest and are responsible for the contents of this report.

## AUTHOR CONTRIBUTIONS

Yaodong Zhou involved in the conceptualization, methodology and investigation of the document; wrote; edited; and visualized the original draft of the manuscript. Yixin Zhang involved in the conceptualization and methodology of the study, wrote, reviewed and edited the manuscript.

## Data Availability

The data that support the findings of this study are available from the corresponding author upon reasonable request.
